# Retinal Microvascular Abnormalities and Systemic Arterial Stiffness Are the First Manifestation of Cardiovascular Abnormalities in Patients with Untreated Moderate to Severe Obstructive Sleep Apnoea and with Low to Intermediate Cardiovascular Risk—A Pilot Study

**DOI:** 10.3390/biomedicines10102669

**Published:** 2022-10-21

**Authors:** Swathi Seshadri, Hala Shokr, Doina Gherghel

**Affiliations:** 1 Vascular Research Laboratory, College of Health and Life Sciences, Aston University, Birmingham B4 7ET, UK; 2 Pharmacy Division, Faculty of Biology, Medicine and Health, University of Manchester, Manchester M13 9PL, UK; 3 Division of Cardiovascular Sciences, University of Manchester, Manchester M13 9PL, UK

**Keywords:** obstructive sleep apnoea, cardiovascular risk, microvessels, vascular function

## Abstract

This study aimed to investigate macro- and microvascular function parameters and their relationship with known markers of cardiovascular risk in patients with untreated moderate to severe obstructive sleep apnoea (OSA). Fourteen patients with moderate to severe OSA and fourteen controls were included in the present study. General assessments included BMI, systemic blood pressure (BP) and circulating markers for oxidative stress and endothelial function. Additional assessments included 24 h BP and heart rate monitoring, as well as the assessment of heart rate variability. Macro- and microvascular assessments included augmentation index, carotid intima-media thickness, brachial artery flow-mediated dilation, as well as various retinal microvascular function assessments, using the Dynamic Retinal Vessel Analyzer. All participants completed the Short Form Health Survey, Functional Outcomes of Sleep Questionnaire, and Epworth Sleepiness Scale. The results show that, in comparison to controls, BMI (*p* = 0.003) and AIx (*p* = 0.025) were significantly higher in the OSA group. There was, however, no significant difference between groups with regard to other measured systemic general, vascular and circulatory parameters (all *p* > 0.05). Nevertheless, the retinal microvascular function showed various alterations in the OSA patients, including a delayed reaction time in response to flicker (*p* = 0.047), as well as a decreased dilation amplitude (*p* = 0.004), dilation slope (*p* = 0.004), and post-flicker constriction (*p* = 0.015). In addition, the observed Slope_AD_ alterations correlated negatively with BMI values only in the OSA group (r = −0.46, *p* = 0.045). In conclusion, individuals with untreated moderate to severe OSA but without overt CVD, exhibit signs of increased arterial stiffness and retinal microvascular dysfunction, which can be early indicators for future vascular complications.

## 1. Introduction

Given the intimate associations between blood flow regulation and sleep–wake cycle, sleep disorders are increasingly becoming recognized as important risk factors for the development and progression of cardiovascular disease (CVD) [1].

The mechanisms by which sleep disturbances adversely affect cardiovascular structure and function are thought to be multiple. Indeed, OSA is associated with increased sympathetic excitation, vascular endothelial dysfunction and metabolic dysregulation, as well as with increased oxidative stress and inflammation induced by intermittent hypoxia [2], all important risk factors for CVD. Moreover, it has been demonstrated that treatments of OSA improve not only patient-reported outcomes (such as sleepiness, quality of life, and mood) but also reduce nonfatal cardiovascular events, as well as CVD-related mortality [2].

Apnoea–hypopnea index (AHI) represents the most common diagnostic metric used to characterize the severity of OSA; nevertheless, this parameter does not strongly predict adverse cardiovascular health outcomes related to this disease [1]. In addition, assessments of various established blood biomarkers give contradictory results when it comes to the characterization of cardiovascular risk in OSA [3]. Due to the early presence of vascular endothelial dysfunction in this disease [2,3,4,5], it is possible, however, that the non-invasive assessments of vascular function could offer a better CVD risk assessment and diagnosis alternative. Indeed, even in the absence of any other co-morbidities, chronic intermittent exposure to hypoxia, as it that encountered in OSA, can, in time, cause endothelial dysfunction and augmented vascular constriction that, finally, culminate into CVD. This dysfunction can be assessed early in the course of the disease; therefore, functional changes at both macro- and microcirculatory levels, could provide good predictions for future development of various vascular complications in individuals at risk, including in patients with OSA without overt CVD [4,5,6,7,8].

Therefore, in the present study, we have investigated macro- and microvascular function parameters and their relationship with known circulatory markers of cardiovascular risk in patients with untreated moderate to severe OSA with low-to-moderate CVD risk, in comparison with age-matched controls.

## 2. Materials and Methods

### 2.1. Study Participants

Prior to study initiation, ethical approval was sought and received from University Hospitals Birmingham NHS Foundation Trust ethics committee, as well as Aston University Research Ethics Committee. Written informed consent was received from all participants prior to study enrolment and all study procedures were designed and conducted in accordance with the tenets of the Declaration of Helsinki.

All patients referred to the Birmingham Heartlands Respiratory Physiology and Biomedical Research Centre Sleep Unit (Birmingham, UK) for the possibility of OSA, and who underwent overnight Polysomnography (PSG) assessment were considered for this study. A team of sleep physiologists examined all recruited study patients and only those classified as having OSA, where CPAP therapy was indicated as the first-line treatment, were considered for the study. Patients who had undergone the overnight PSG but who were not considered to be suffering from OSA were included as study controls.

All participants were provided with detailed information about the study and allowed at least 24 h to consider their enrolment. Participants who provided consent were then requested to attend a study assessment at the Vascular Research Laboratory (Aston University, Birmingham, UK), prior to the initiation of OSA treatment. The diagnosis of OSA and treatment assignment was masked from the research team that performed the measurements and data analyses.

Study exclusion criteria were defined as the positive diagnosis of CVD, cerebrovascular disease, peripheral vascular disease, severe dyslipidaemia (defined as plasma triglycerides > 6.00 mmol/L or cholesterol levels > 7.00 mmol/L), diabetes, as well as other metabolic disorders or chronic diseases that required treatment. Individuals treated for systemic hypertension as well as those using any vasoactive medications such as dietary supplements containing vitamins or antioxidants and bronchodilators were also excluded from the study. Potential participants were also screened for ocular diseases and were excluded from the study if they had a refractive error of more than ±3DS and more than ±1DC equivalent, intra-ocular pressure (IOP) greater than 21 mmHg, cataract, or any other media opacities, as well as history of intra-ocular surgery or any form of retinal or neuro-ophthalmic disease affecting the ocular vascular system. Individuals with sings of hypertensive retinopathy at the initial fundus examination were also excluded.

### 2.2. Polysomnography

All PSG assessments undertaken at the sleep clinic included 2-channel Electroencephalography (EEG), 2-channel Electro-oculography (EOG), 1-channel submental electromyography (EMG), respiratory and abdominal movements via chest and abdominal belts, nasal pressure via pressure sensor, and oximetry using a finger oximetry probe. Sleep stages and respiratory parameters were scored by the attending sleep physiologist according to standard American Academy of Sleep Medicine guidelines [9]. The AHI was referred to as the average number of apnoea events (complete cessation of airflow for at least 10 s) and hypopneas (reduction in airflow of at least 30% accompanied by at least a 4% blood oxygen desaturation in the preceding 30 s, and a reduction in chest wall movement and/or arousal) per hour of sleep. Patients with an AHI > 15 or greater per hour were defined as having moderate to severe OSA and were included in the study. Individuals with an AHI < 5 per hour were defined as not having OSA and were included into the study as controls. Patients with mild OSA (AHI of 5 ≤ AHI < 15 per hour) were excluded from the study.

Other study exclusion included history of smoking; unstable CVD such as CAD, valvular heart disease and heart failure; chronic lung disease such as chronic pulmonary obstructive disease (COPD) and bronchiectasis; renal failure, previous history of OSA treatment such as CPAP therapy; other sleep disorders such as insomnia, narcolepsy, and shift-work related sleepiness; and history of vasoactive medications known to affect vascular and or endothelial function.

### 2.3. Sleep Questionnaires

All study participants completed the general health history questionnaire, as well as subjective quality of life (QoL) and sleep questionnaires as detailed below.

#### 2.3.1. General Health and Quality of Life Questionnaire: Short Form-36 (SF-36^®^)

The SF-36^®^ is a validated, 36-item questionnaire used as measure of subjective health status and quality of life (QoL). The instrument provides an 8-scale, with 36 self-reported health measures [10].

The SF-36 is a 36-item scale, measures eight domains of health status: physical functioning (10 items); physical role limitations (four items); bodily pain (two items); general health perceptions (five items); energy/vitality (four items); social functioning (two items); emotional role limitations (three items) and mental health (five items). A scoring algorithm is used to convert the raw scores into the eight dimensions listed above. The scores are transformed to range from zero where the respondent has the worst possible health to 100 where the respondent is in the best possible health.

#### 2.3.2. Functional Outcomes of Sleep Questionnaire (FOSQ)

The FOSQ is a 30-item self-report questionnaire designed to measure the impact of excessive sleepiness on multiple activities of daily living, conceptually defined as functional status, and a number of studies support the validity and reliability of FOSQ as an outcome measure in clinical trials [11].

The FOSQ comprises five dimensions: activity level, vigilance, intimacy and sexual relationships, general productivity, and social outcome. Each of these dimensions is rated on a 4-point scale (no difficulty to extreme difficulty). The questions pertaining to sexual intimacy and relationships were excluded from the questionnaire in the present research due to the personal nature of the questions, and possible pitfalls associated with non-response to these items [12].

### 2.4. General Investigations

Standard anthropometric measures of height and weight were recorded to determine body mass index (BMI = weight/height^2^). Systolic blood pressure (SBP), diastolic blood pressure (DBP), and heart rate (HR) were measured using an automatic Blood Pressure monitor (UA-767; A&D Instruments Ltd., Abingdon UK) [13] to determine mean arterial pressure (MAP = 2/3 DBP + 1/3 SBP) [14]. Intraocular pressure (IOP) readings were obtained using non-contact tonometry (Pulsair; Keeler Ltd., Winsor, UK).

### 2.5. Blood and Urine Analyses

Blood and plasma samples drawn from the antecubital fossa vein were assessed immediately for fasting glucose (GLUC), triglycerides (TG), total cholesterol (T-CHOL), high-density lipoprotein cholesterol (HDL-C) and glycated haemoglobin (HbA1c) using the Reflotron Desktop Analyzer (Roche Diagnostics, Welwyn Garden City, UK). Low-density lipoprotein cholesterol (LDL-C) values were calculated as per the Friedewald equation [15].

These variables, in addition to the above parameters, were used to calculate the Framingham Risk Score (FRS) for each individual [16]. Absolute CVD risk percentage over 10 years was classified as low risk (<10%), intermediate risk (10–20%) and high risk (>20%) [17].

Additional set of urine samples were also collected and sent to the pathology unit for assessment of albumin/creatinine ratio (ACR).

### 2.6. Measurement of Glutathione Redox Index

Glutathione recycling assays (oxidized (GSH) and reduced (GSSG)) were also performed, as detailed previously [18]. Briefly, a 30 μL aliquot of EDTA blood was pre-treated with 33.3 μL of 100 mg/mL 5-sulfosalicylic acid (SSA), 936.7 μL sodium phosphate buffer (pH 7.5) to release GSH via cellular disruption and protein precipitation. The sample was centrifuged at 13,000 rpm for 5 min, and the supernatant was stored at −80 °C for further analyses. Based on previous reports of sample stability, assays were conducted within 2 months of collection [19]. The GSH levels [t-GSH − (2 × GSSG)] and the redox index (defined as the GSH/GSSG ratio) were determined according to an established enzymatic recycling assay [20,21].

### 2.7. Measurement of Nitric Oxide

Fasting venous blood samples were collected in citrated tubes. Samples were centrifuged at 3000 rpm for 15 min. The citrated plasma was then thawed and diluted 1:2 in1X reagent diluent and levels of NO were measured in triplicates using Invitrogen nitric oxide assay kit^®^ (Bender Medsystems GmbH, Vienna, Austria) [7].

### 2.8. Macro- and Microvascular Assessments

#### 2.8.1. Dynamic Retinal Microvascular Function Vessel Analysis

**Figure 1:**
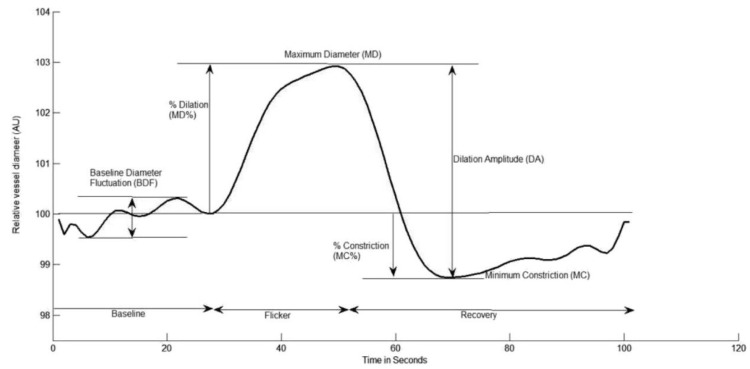
Graphical presentation of the dynamic vessel response profile displaying the parameters calculated and used in analysis. (DA) calculated as (MD-MC). (MD%) calculated as the percent increase from baseline to MD. (MC%) calculated as the percent constriction below baseline following MD.

Retinal microvascular function was assessed using the dynamic retinal vessel analyser (DVA, IMEDOS GmbH, Jena, Germany) in accordance with an established protocol [22] Using a validated in-house algorithm, the following vessel reactivity and time-course parameters were determined: the average baseline diameter and range of maximum and minimum baseline vessel diameters (baseline diameter fluctuation, BDF); the maximum vessel dilation diameter during flicker stimulation expressed as a percentage change relative to baseline diameter (MD%) and the time taken in seconds to reach the maximum diameter (tMD); the maximum vessel constriction diameter during the post-flicker recovery period expressed as a percentage change relative to baseline diameter (MC%) and the time taken in seconds to reach the maximum vessel constriction diameter (tMC); the overall dilation amplitude (DA) calculated as the difference between MD and MC; and the baseline-corrected flicker response (BCFR) used to describe the overall dilation amplitude after normalizing for fluctuations in baseline diameters (DA-BDF). In addition, the arterial (A) and venous (V) dilation slopes (SlopeAD/VD = (MD − baseline diameter)/tMD) and constriction slopes (SlopeAC/VC = (MC − MD)/tMC) were calculated (Figure 1) [6,23].

#### 2.8.2. Pulse Wave Analysis (PWA)

PWA was conducted in accordance with an established protocol using the validated SphygmoCor device (AtCor Medical/PWV Medical Pty Ltd., Sydney, Australia) [24]. The patient’s radial pulse was first located just below the wrist creases at the base of the thumb and the SphygmoCor transducer or high-fidelity pressure sensor was flattened over this site with slight pressure to generate a signal representative of the intravascular pulse in the radial artery. Reasonable confidence in readings was gained when pressure waves were consistent from beat to beat and with characteristics to be expected in the artery (sharp upstroke to the first systolic peak, sharp cleft and near-exponential pressure decay in late diastole). The pulsatile radial waveform was then calibrated against SBP and DBP readings by the in-built software, and mathematically transformed using a transfer function to reconstruct the aortic waveform from which a range of central cardiovascular parameters can be derived.

#### 2.8.3. Carotid-Intima-Media Thickness (c-IMT)

**Figure 2:**
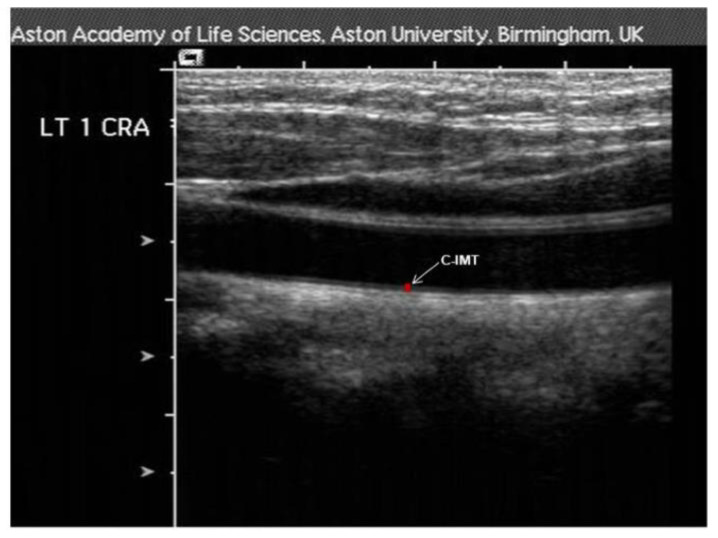
Ultrasound image of carotid intima-media measurement site. C-IMT: carotid intima-media thickness.

c-IMT measurements were conducted in accordance with a well-established protocol [25]. Using high-resolution B-mode ultrasonography (Siemens, Acuson Sequoia^®^, Camberley, UK). The patients were in a resting position with their head turned towards one side and neck slightly extended. Typically, high-resolution ultrasound imaging reveals a double-line pattern in the inner vessel lumen (Figure 2). c-IMT measurements were then taken from central region of the inferior wall of the artery using the in-built software calliper system at a site proximal to the bifurcation.

#### 2.8.4. Flow-Mediated Dilation

In accordance with previously published guidelines for assessment [26]. The patient was positioned supine and following a brief acclimatization period, the arm was extended in a comfortable position and the brachial artery was imaged above the antecubital fossa in the longitudinal plane using high-resolution ultrasonography with a 7 mm, 8 MHz linear-array transducer (Siemens; Acuson Sequoia^®^, Camberley, UK). A clear segment of the vessel with visible anterior and posterior intimal interfaces between the lumen and vessel wall was then selected for continuous imaging. Vessel diameters were continually recorded from the selected region of interest using a specialised wall-detection and artificial neural networking software (VIA^®^ Software, UK). Based on published recommendations, a baseline image was acquired for 2 min, following which a BP cuff positioned at the forearm was inflated to a supra-systolic pressure (50 mmHg above systolic) for 5 min; effectively occluding blood flow through the brachial artery, inducing hypoxia, and causing dilatation of downstream resistance vessels. Thereafter, image acquisition was carried out through the cuff inflation phase and continued for an additional 2 min post-cuff deflation (hyperaemia).

### 2.9. Statistical Analysis

All statistical analyses were performed using Statistica^®^ software (StatSoft Inc., Version 16, Tulsa, OK, USA). Differences in mean values between groups were compared by independent samples *t*-test for continuous variables. Multivariate analysis was performed to investigate possible influences of age, gender, BMI, BP, and circulating markers on the measured variables. Differences between groups in retinal and systemic vascular function parameters were computed by *t*-test or analysis of covariance (ANCOVA) where applicable. Comparisons of retinal function parameters for individual flicker periods were carried out by two-factor repeated-measures analysis of variance (ANOVA). Statistical significance was set at *p* < 0.05.

#### Sample Size Calculation

All sample size calculations were performed using the G*Power software (University of Kiel, Version 3.1.6, Kiel, Germany). With regard to retinal vascular function, the measurement parameters and polynomial regression fitting methods used in this thesis have not previously been reported in OSA patients. Sample size calculations were therefore based on previous studies, which share similar protocols with that of the present study. On the basis of functional retinal studies using DVA, a change of 30% with an SD of 2.5% has been shown to be clinically significant [27]. With regard to FMD, a brachial reactive hyperaemic response of 10.93% with a standard deviation of 2.59% is considered normal on the basis of previous research and an approximately 30% alteration in this response has been shown to be clinically significant in OSA patients [28]. It was anticipated that t-tests and repeated measures ANOVA would be required in this study; therefore, in order to provide a statistical power of at least 80% with an alpha-level set at 0.05, it was estimated that a sample size of 10–14 participants per group would be required (14 DVA within/between ANOVA, 10 FMD *t*-test).

## 3. Results

### 3.1. General and Respiratory Measurements

Of the 31 participants originally recruited to the study, 3 participants were excluded based on poor-quality DVA recordings. The remaining 28 adults (18 men and 10 women), aged between 43 and 69 years were selected for the final analysis, and included 14 OSA and 14 control participants (based on AHI values). Table 1 provides a summary of general characteristics, clinical data and systemic parameters.

BMI levels were significantly higher in the OSA group compared with controls (*p* = 0.003). Interestingly, there were, however, no other significant group differences in all the other general and circulatory measures, including their overall FRS (all *p* > 0.05).

With regard to PSG, as expected, AHI values were significantly higher in the OSA group (*p* < 0.001, Table 1). However, there were no significant differences in the general health, QoL and sleep quality assessments between the study groups (all > 0.05).

After correcting for influential variables, there were also no significant differences in c-IMT scores or the assessed FMD parameters (all *p* > 0.05). However, AIx values were higher in OSA subjects in comparison to controls (*p* = 0.025) (Table 2).

### 3.2. Retinal Vascular Function

**Figure 3:**
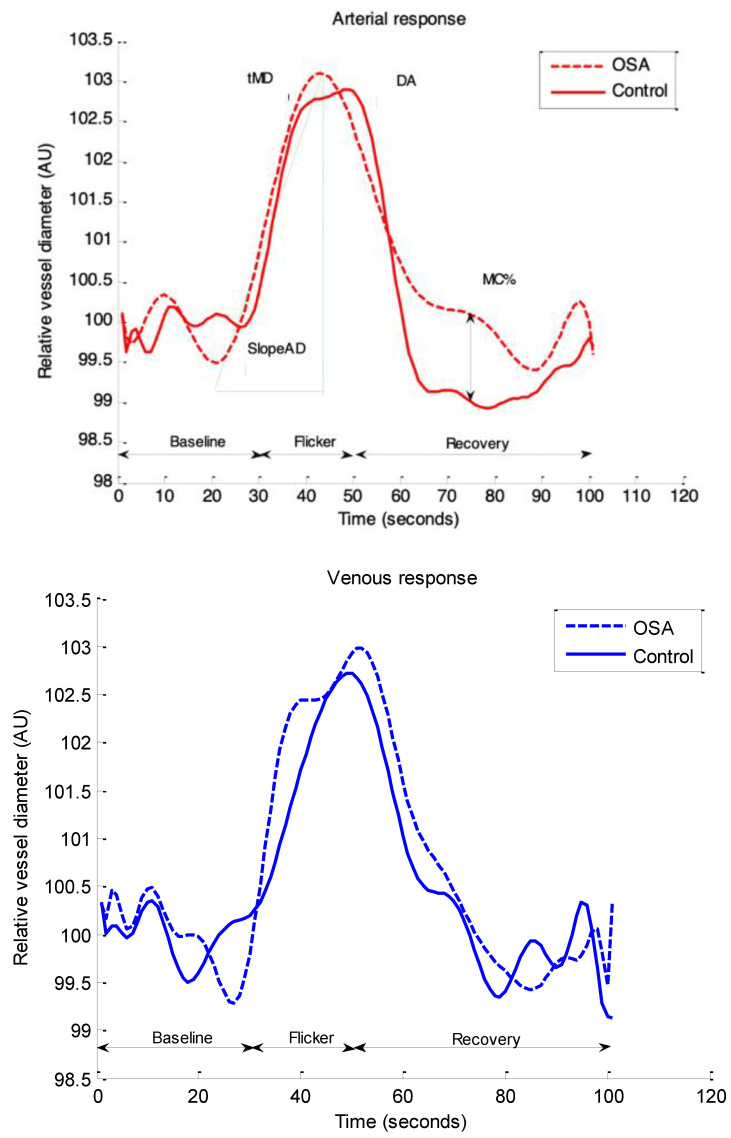
Group comparisons of averaged retinal vascular response profiles. Abbreviations: AU, arbitrary units; DA, dilation amplitude; tMD, time to maximal dilation. diameter; MC% percent constriction; Slope_AD_, arterial dilation slope.

Retinal vascular function parameters were averaged across three flicker cycles with the artery and vein regarded separately (Table 3). After correcting for influential covariates identified in multiple regression models, arterial tMD (*p* = 0.047) was longer, while DA (*p* = 0.004), Slope_AD_ (*p* = 0.004), and MC% (*p* = 0.015) were decreased in the OSA group compared with controls (Figure 3). No other group differences were identified in any of the other measured averaged retinal arterial and venous parameters (all *p* > 0.05 data for the venous parameters are not shown).

### 3.3. Correlation Analyses

In within-group correlations, BMI significantly and negatively correlated with averaged Slope_AD_ in OSA subjects (r = −0.46, *p* = 0.045). A similar trend was, however, not observed in the control group (*p* > 0.05).

## 4. Discussion

The results of this study indicate that in our sample of OSA subjects with untreated moderate to severe sleep apnoea, despite a low to intermediate cardiovascular risk as determined using FRS, there were detectable signs of systemic arterial stiffness and retinal microvascular dysfunction. These abnormalities could represent the first manifestation of CVD in this type of patients.

Previous studies have reported retinal abnormalities of a structural nature in patients with OSA with high CVD risk [28,29]. In the present study, however, despite having a high BMI, our OSA patients had a low to intermediate CVD risk as determined by FRS. In this group of patients, despite the lack of overt CVD, we have found various changes in the retinal microvascular function, in the forms of greater fluctuations in arterial baseline diameter, delayed reaction times, decreased dilation post-flicker as well as abatements in the re-establishment of baseline diameter. Baseline diameter fluctuation represents a factor that, although recognized as important [29], is not commonly discussed in the literature. However, its abnormalities could indicate the existence variations in the vascular tone, vascular stiffness or the presence of various ischemic and hypoxic changes at the vascular level. Indeed, our OSA patients also exhibited increased systemic arterial stiffness as assessed using PWA and, although the observed attenuations in retinal vascular function appeared to be independent of this parameter, it cannot, however, be excluded that the general vascular stiffness played some role in our findings. This possibility is further supported by the observation that our patients also exhibited decreased dilation and constriction of the retinal vessels during and post-flicker stimulation, a definite sign for the lack of elasticity of these microvessels.

The intermittent hypoxic events, associated with OSA, are known to induce an increase in the free radical production [30,31] which, in turn, is associated with impaired NO bioavailability and, therefore, vascular dysfunction [32,33,34]. At the retinal level, although alterations in the metabolic demand and neurovascular coupling are most largely involved in the retinal vascular response to flickering light [35], a compromised NO homoeostasis is also known to play a role in any vascular dysfunction recorded at this level. In our study, however, there were no differences in either the measured anti-oxidative parameters or in the NO levels between the two study groups. Nevertheless, these measurements will assess only the basal levels of the above-mentioned parameters. It is possible that the increased needs for NO secretion during flicker stimulation were not met in our OSA patients. In addition, another explanation could be offered by a possible rapid NO removal post-stimulation [36]. However, all these hypotheses need to be tested in further studies.

An additional finding of this study was the attenuation of retinal arterial vasoconstrictive responses following flicker in OSA subjects, which is comparable to what has previously been reported in other conditions associated with hypoxia [37]. However, it is possible that our observation could also occur as a consequence of a certain degree of general vascular stiffness. Nevertheless, the observed simultaneous attenuations in the dilation capacity could signal, in fact, that our findings are mostly due to the presence of a true vascular dysfunction and not to a generalize vascular stiffness, age-related or otherwise.

An interesting observation was the correlation between BMI values and Slope_AD_ in patients with OSA. We have previously published that otherwise healthy overweight individuals present with signs of microvascular functional impairment, as well as, with an increased circulatory plasma markers of endothelial dysfunction when compared with lean individuals [38,39]. Our conclusions were that these findings can occur due to either early atherosclerosis, increased arterial stiffness, or reduced NO bioavailability to peripheral tissues [40]. In addition, microvascular dysfunction in patients with an increased BMI can be triggered in areas with perivascular adipose tissue (PVAT) by subclinical inflammation mediated via adipokines/cytokines and infiltrating macrophages [41]. Nevertheless, these molecules can also trigger remote inflammatory effects withy potential vascular changes as far as the retinal circulation, usually devoid of PVAT, therefore, resulting in abnormal vascular function at this level [42]. Further research is necessary to shed more light into these possible contributions to our observations.

## 5. Conclusions

Patients with untreated moderate to severe OSA and with low to intermediate CVD risk exhibit significant systemic arterial stiffness as well as attenuations in retinal vascular function. Functional retinal assessments could therefore be useful for early vascular screening in these patients as well as in other groups at-risk for CVD.

## Figures and Tables

**Table 1:** Abbreviations: BMI, body mass index; SBP, systolic blood pressure; DBP, diastolic blood pressure; HR, heart rate; MAP, mean arterial pressure; IOP, intraocular pressure; OPP, ocular perfusion pressure; GLUC, glucose; TG, triglycerides; CHOL, total cholesterol; HDL-c, high-density lipoprotein cholesterol; LDL-c, low-density lipoprotein cholesterol; GSH, reduced glutathione; GSSG, oxidized glutathione; tGSH, total GSH; Redox index, GSH:GSSG; ET-1, endothelin-1; NO, nitric oxide; FRS, Framingham risk score; AHI; apnoea/hypopnea index; n/h, number of episodes per hour.

	Mean (SD)		
Variable	Group (1)	Group (2)	*p*-Value (*t*-Test)
	OSA	Control	
N	14	14	-
Gender	10M/4W	9M/5W	0.987
Age (years)	59 (7)	60 (9)	0.890
BMI (kg/m^2^)	34.09 (6.50)	28.04 (3.35)	0.003 *
SBP (mmHg)	130 (15)	125 (15)	0.690
DBP (mmHg)	80 (5)	80 (7)	0.968
HR (bpm)	83 (8)	77 (11)	0.480
IOP (mmHg)	16 (3)	15 (2)	0.650
GLUC (mmol/L)	4.65 (0.55)	4.61 (0.63)	0.927
TG (mmol/L)	2.12 (1.50)	1.32 (0.50)	0.094
CHOL (mmol/L)	5.25 (1.32)	5.21 (0.80)	0.947
HDL-C (mmol/L)	1.23 (0.50)	1.21 (0.35)	0.950
LDL-C (mmol/L)	3.00 (1.05)	3.30 (0.80)	0.475
tGSH (µmol/L)	975 (350)	1200 (552)	0.100
GSH (µmol/L)	838 (320)	1154 (492)	0.085
GSSG (µmol/L)	75 (22)	87 (45)	0.480
NO (µmol/L)	6.50 (3.85)	5.40 (2.00)	0.285
FRS (%)	10.00 (4.50)	8.85 (6.00)	0.700
AHI (n/h)	42 (24)	2 (2)	<0.001 *

* Significant *p*-values are indicated in bold where *p* < 0.05 was considered significant.

**Table 2:** Abbreviations: c-IMT, carotid intima-media thickness; R, right; L, left; PWA, pulse-wave analysis; AIx, augmentation index; FMD, flow-mediated dilation; AD, average baseline brachial diameter; MDhyperaemia, maximum brachial diameter during hyperaemia; FMDED, endothelium-dependent flow-mediated dilation. * Significant *p*-values are indicated in bold where *p* < 0.05 was considered significant.

	Mean (SD)		
Parameter	Group (1)	Group (2)	*p*-Value (*t*-Test/ANCOVA)
	OSA	Control	
c-IMT			
R-IMT (cm)	0.75 (0.15)	0.70 (0.11)	0.300
L-IMT (cm)			
PWA			
AIx	25 (12)	19 (12)	0.025 *
FMD			
FMD_ED_ (%)	12.35 (10.50)	14.00 (11.25)	0.780

**Table 3:** Abbreviations: ANOVA, analysis of variance; ANCOVA, analysis of covariance; Baseline, baseline diameter; BDF, baseline diameter fluctuation; BCFR, baseline corrected flicker response; DA, dilation amplitude; MD%, percent dilation; MC%, percent constriction; tMD, reaction time to MD; tMC, reaction time to MC (time between MD and MC); Slope_AD/VD_, slope of arterial/venous dilation; Slope_AC/VC_, slope of arterial/venous constriction. * Significant *p*-values are indicated in bold where *p* < 0.05 was considered significant.

	Mean (SD)		
DVA Parameter	Group (1)	Group (2)	*p*-Value
	OSA	Control	
***Arteries:***			
Baseline	99.55 (0.50)	99.80 (0.20)	0.901
BDF	5.77 (2.31)	5.85 (2.35)	0.888
BCFR	−1.09 (3.54)	−0.60 (2.36)	0.449
DA	4.89 (2.00)	6.50 (2.00)	0.004 *
MD%	3.65 (2.00)	3.70 (2.48)	0.780
MC%	−0.90 (3.54)	−2.25 (3.00)	0.015 *
tMD (seconds)	23 (10)	18 (10)	0.047 *
tMC (seconds)	29 (10)	29 (12)	0.964
Slope_AD_	0.22 (0.21)	0.43 (0.40)	0.004 *
Slope_AC_	−0.20 (0.10)	−0.27 (0.15)	0.410

## Data Availability

The data presented in this study are available on request from the corresponding author. The data are not publicly available due to ethical regulations.
